# A systematic review of proximal humerus fractures and associated vascular injuries

**DOI:** 10.1016/j.jvsvi.2024.100065

**Published:** 2024

**Authors:** Jenna Shepherd, Athanasios Saratzis, Coral Pepper, Harvinder Singh, Sarah Jane Messeder

**Affiliations:** a Department of Orthopaedic Surgery, Leicester General Hospital, University Hospitals of Leicester, Leicester, UK; b Department of Cardiovascular Sciences, University of Leicester, NIHR Leicester Biomedical Research Centre, Glenfield Hospital, Leicester, UK; c Leicester Vascular Institute, University Hospitals of Leicester, Glenfield Hospital, Leicester, UK; d Library and Information Services, University Hospitals of Leicester NHS Trust, Leicester Royal Infirmary, Infirmary Square, Leicester, UK

**Keywords:** Humeral fracture, Vascular injury, Trauma

## Abstract

**Objective:**

Proximal humerus fractures are common with a reported neurovascular injury incidence of 0.09% to 5%. This study aimed to synthesize the current evidence on the presentation and management of proximal humerus fractures with associated vascular injury to aid clinical decision-making.

**Methods:**

A systematic review was conducted following Preferred Reporting Items for Systematic Reviews and Meta-Analyses (PRISMA) guidelines (PROSPERO registration: CRD42023393957) to identify articles reporting proximal humerus fractures with associated vascular injury in adults. Study quality was assessed using the Joanna Briggs Institute critical appraisal tools checklist. Outcomes included presentation, fracture classification, type of vascular injury, method of orthopedic and vascular repair, and complications.

**Results:**

A total of 40 articles representing 55 individuals with a fracture were included. Injuries most commonly occurred after a low-energy mechanism such as a fall from a standing height (n = 32, 58%). The presentation of ischemia included cool limb (n = 29, 53%), pallor (n = 21, 38%), prolonged capillary refill (n = 7, 13%), and an absent or reduced pulse (n = 47, 85%). Concomitant neurological injury was reported in 30 cases (55%) and fracture dislocations were reported in 17 cases (32.7%). Fracture classification was variable; however, when all recorded fracture patterns were described in terms of 2-, 3-, or 4-part fractures, these represented 49% (n = 27), 24% (n = 13), and 18% (n = 10), respectively. Fracture management preceded vascular repair in 30 (55%). Orthopedic management was primarily performed by open reduction internal fixation or wire fixation (n = 33, 60%) and hemiarthroplasty (n = 11, 20%). Isolated arterial injury was the most common vascular injury (n = 52, 95%). Arterial injuries were primarily repaired by an interposition graft (n = 21, 38%), primary repair (n = 11, 20%), or conservative management (n = 9, 16%). Complications such as amputation, compartment syndrome, avascular necrosis, and metalwork failure were reported in 13 cases.

**Conclusions:**

Proximal humerus fractures with associated vascular injuries occur most commonly in the older adults after low-energy mechanisms such as a fall from a standing height. A high index of suspicion is needed as not all injuries present with classical ischemic symptoms, and these injuries carry significant associated morbidity. (JVS-Vascular Insights 2024;2:100065.)

Proximal humerus fractures represent 5% to 6% of all adult fractures and are the third most common nonvertebral fractures in those over the age of 65 years.^1,2^ They are associated with significant morbidity, functional disability, and socioeconomic impact, with a mortality rate of 3.2%.^3^ Despite this, consensus on the management of proximal humerus fractures is lacking with insufficient evidence from major randomized controlled trials between surgical techniques and whether they should be managed operatively or nonoperatively.^4–6^

Associated neurovascular injury is rare in proximal humerus fractures, with varying reported incidence of between 0.09% and 5%.^1,7^ Unfortunately, neurovascular injury is often missed in individuals presenting with minor blunt trauma.^8^ This is significant given that these injuries are linked to a higher mortality, prolonged hospital stay, and increased hospital cost.^7^ Guidelines suggest that ischemic limb revascularization should occur within 4 hours from injury, and therefore, prompt diagnosis is essential.^9^ Data from Alarhayem et al^10^ demonstrated that shorter injury to operating room times decreased amputation rates, with a significant increase in rate after 1 hour.

To reduce morbidity, the surgical management of vascular injury requires the use of shunts where rapid definitive revascularization cannot be achieved before fracture fixation. Guidelines also suggest that definitive repair or direct interposition grafts are preferable to bypass graft for the treatment of vascular injury associated with fractures.^9^ Proximal humerus fracture patterns are variable, and several classification systems are used to characterize these.^11,12^ Surgical options for fracture management include fixation with k-wire, plate, or intramedullary nail and replacement with hemiarthroplasty or reverse shoulder arthroplasty.^4^

The aim of this systematic review is to synthesize the current evidence on the presentation, investigation, and management of proximal humerus fractures with associated vascular injury in order to aid clinical decision-making. We aimed to determine fracture patterns associated with vascular injury, clinical examination findings, imaging used for diagnosis, method of fracture fixation and vascular repair used, and postoperative outcomes.

## Methods

A systematic review was conducted according to the Preferred Reporting Items for Systematic Reviews and Meta-Analyses (PRISMA)^13^ and registered with the International Prospective Register of Systematic Reviews (PROSPERO; CRD42023393957).

### Search strategy

Studies were identified by searching Ovid MEDLINE, Embase, CINAHL, Cochrane Database of Systematic Reviews, and Cochrane Central Register of Controlled Trials from database inception until February 1, 2023. The search strategy (Supplementary Fig, online only) was developed in conjunction with a clinical librarian (C.P.) and used the terms shoulder fracture, humeral fracture, vascular/blood vessel injury or damage, amputation, revascularization, and limb salvage. No limitation on language or date was applied. Search results were combined into Covidence (Covidence systematic review software; Veritas Health Innovation) and all duplicates removed.

### Study selection

Two independent reviewers (J.S. and S.J.M.) screened study title and abstracts against inclusion and exclusion criteria (Supplementary Table I, online only). Full papers were then accessed and screened independently (J.S. and S.J.M.) with conflicts settled by a third independent screener (A.S.). Inter-rater reliability for screener agreement was reported with calculation of Cohen’s kappa.

### Quality assessment and data extraction

Risk of bias was assessed using the Joanna Briggs Institute critical appraisal tools checklist^14^ (Supplementary Tables II and III, online only), and conflicts of interest statements were collected. Data extraction was carried out by 1 reviewer (S.J.M.). Data extracted included year, country, and setting of the study; inclusion/exclusion criteria; patient demographics; patient history and clinical examination findings; imaging modalities used; fracture type and vascular injury; operative technique; and patient outcome. A full narrative synthesis was undertaken.

## Results

A PRISMA flow diagram was used to summarize the flow of studies (Fig). Cohen’s kappa for title and abstract screening and full text screening was 0.86 and 0.73, respectively, representing almost perfect and substantial agreement.^15^ In total, 40 studies were included within the systematic review: 29 case reports and 11 case series. For those that were case series, only individuals meeting the inclusion criteria were included in the systematic review.

### Description of population

A total of 40 studies representing 55 individuals were included in the systematic review. The median age was 68 years (interquartile range: 52.5-79 years). There were similar numbers of females (n = 26, 47%) and males (n = 25, 45%). Two studies did not report on sex.^16,17^

### Clinical presentation

Table I summarizes the clinical presentation of individuals included per study. The most common mechanism of injury was a fall at a standing height (n = 32, 58%),^16–18,20,21,24,26,28–31,34,35,37–41,46–52,54,55^ followed by high-energy blunt trauma (n = 21, 38%).^16,17,19,22,25,27,33,35,36,41–46,51,54^ Most individuals presented with a cool limb (n = 29, 53%)^16,17,22,24,26–28,33–37,39–42,44,46,47,50,52–55^ followed by pallor (n = 21, 38%)^16,17,19,20,26–28,30,33,36,40–42,47,48,50,52,54,55^ and swelling (n = 9, 16%).^16,30,35,37,40,41,43,51,54^ Concomitant neurological injury was reported in 30 cases (55%), with 13 having alteration in both motor and sensory function.^17,20,22,25,27,31,36,45,47,51,53,54^ Only one individual sustained an open fracture; all other injuries were closed in nature.^46^ On examination, most individuals had an absent or reduced pulse (n = 47, 85%). Capillary refill time was increased in seven cases (13%).^17,26,27,33,48,52,54^ Nearly a third of cases had an absent or reduced Doppler signal (n = 17, 31%).^17,19–21,23,24,26,27,31,35,39,45,49,52,55^ Hemodynamic instability was reported in only one case (2%).^45^

### Imaging

All fractures were diagnosed by plain film except one case, which was diagnosed on computed tomography (CT) imaging.^54^ Vascular injury was diagnosed by CT angiogram in 20 cases (36%),^20,22–26,28,30,32,42,43,46,48,49,54^ digital subtraction angiography in 17 cases (31%),^16,17,21,31,33–35,37,38,40,41,45,47,51^ and arterial duplex in two cases (4%).^52,54^ Sixteen cases (29%) did not report any preoperative vascular imaging.^17–19,26,27,29,35,36,39,41,44,50,51,53,55^ Five (11%) of these cases went on to receive intraoperative imaging in the form of digital subtraction angiography.^26,35,39,40,55^

### Fracture pattern

Table II summarizes the different fracture patterns reported. The fracture classification system used to describe the fracture pattern varied, including Neer (n = 26, 47%),^18,20–22,25,27–30,32,33,37–40,43,46,48–51,53,55^ Arbeitsge-meinschaft für Osteosynthesefragen (n = 3, 5%),^26,30^ and basic anatomical description of site of fracture (n = 21, 38%). Fracture type was not recorded in 9% (n 5) of cases.^23,24,40,41,51^ For the purposes of simplification, however, when all recorded fracture patterns were described in terms of 2-, 3-, or 4-part fractures, these represented 49% (n = 27), 24% (n = 13), and 18% (n = 10), respectively. The majority of fractures were described as displaced, whereas three (5%) were minimally displaced^24,45,52^ and six (11%) did not report degree of displacement.^20,23,36,41,53^ The humeral head was dislocated in 17 (31%)^17,20,21,26,38,39,46,22,27,44,36,53,32^ and subluxed in one case (2%).^34^

### Surgical approach

A deltopectoral approach was most commonly used (n = 28, 51%). Three were managed through an anterolateral approach (5%),^23,42,51^ two through subclavicular approach,^20,21^ and one through a brachial approach.^17^ A total of 21 (38%) cases did not report the surgical approach. Most cases underwent fracture fixation followed by vascular repair (n = 30, 55%), 16 (29%) underwent vascular repair followed by fracture fixation,^16,17,21,22,26,28–32,35,39–41,51,53^ isolated fracture fixation was carried out in four cases (7%),^33,46,54^ isolated vascular repair in two cases (4%),^37,50^ fracture reduction in only one case (2%),^36^ conservative management in one case (2%),^36^ and one case (2%) started with vascular repair, then fracture fixation, and finally further vascular repair.^46^

### Fracture fixation

A summary of fracture management per individual included can be found in Table II. All but three cases (5%) underwent the operative management of the fracture.^16,37,52^ Open reduction internal fixation or wire fixation was the most common operative fracture management (n = 33, 60%), followed by hemi-arthroplasty (n = 11, 20%).^17,22,26,29,34,35,38,46,48^ Specific operative fracture management was not specified in 4% of cases (n = 2).^42,50^ Adjunct procedures included one biceps tenotomy^22^ and one coracoid osteotomy.^49^

### Vascular repair

Table II summarizes the type of vascular repair per individual included. Two cases (4%) reported both arterial and venous injuries^17,22^ and one (2%) an isolated venous injury.^53^ Venous injuries were either ligated^22,53^ or received primary repair.^17^ All other vascular injuries were arterial in origin. Arterial injuries were primarily treated with an interposition graft (n = 21, 38%) with preference for venous over prosthetic conduit.^16–19,23,27,32,34,35,37,40–42,46,48,54,55^ Two interposition vein grafts were converted intraoperatively to interposition prosthetic grafts due to caliber mismatch^18^ or poor flow.^19^ Primary repair was carried out in 11 cases (20%).^17,22,24,25,35,38,47,50,51^ Nine cases were managed conservatively,^33,36,43–46,52,54^ five received endovascular management,^26,28,29,46^ three underwent patch repair,^39,49,51^ two underwent a vein bypass,^20,21^ two were ligated,^31,40^ and one received a prosthetic bypass.^30^ Adjunct procedures included thrombectomy (n = 8, 15%)^22,25–27,38,48,49,55^ and clot aspiration (n = 1).^50^ The use of a shunt was documented in only one case.^46^

### Length of stay

The total length of hospital stay postoperatively was reported in seven studies.^20,21,29,36–38,42^ The median length of stay reported was 7 days post-operatively, ranging from 2 to 24 days.

### Complications

Table III describes the postoperative outcome of individuals included per study. As mentioned, two interposition vein grafts were changed intraoperatively to an interposition prosthetic graft.^18,19^ A total of 13 postoperative complications were reported.

Metalwork failure (n = 1)^39^ and loss of fracture reduction (n = 1)^35^ were observed 1 month postoperatively, leading to revision of fracture fixation in both cases. Avascular necrosis was evident radiologically in one case at 6-month follow-up imaging, following an initial open reduction internal fixation with plate fixation.^20^ After coracoid osteotomy during the surgical approach, coracoid displacement was observed on plain radiographs at 1-year follow-up in one case.^49^ One case of compartment syndrome was reported.^53^ This occurred several hours after the initial procedure following missed arterial injury that required a thrombectomy and vein bypass, where fasciotomy was not undertaken at initial surgery.

Only one case reported limb loss as a complication where a mid-brachial amputation was undertaken at 12 days following the initial procedure. This was a 3-part proximal humerus fracture with arterial injury that underwent clot aspiration followed by primary repair.^50^ Similarly, persistent deltoid and intrinsic muscle weakness at 3 months postoperatively, requiring brachial plexus exploration, was observed in one case.^44^ Systemic complications were also reported in these injuries, including disseminated intravascular coagulopathy (n = 1)^18^ and mortality (n = 4) secondary to myocardial infarction, acute renal failure, and sepsis.^26,35,40,46^ Reported mortality occurred within 2 weeks postoperatively in all cases before patient discharge. Inpatient mortality was reported; however, overall 30-day and 1-year mortality was not reported in any studies.

### Risk of bias

The risk of bias was assessed according to the Joanna Briggs Institute Critical Appraisal Tools checklist. The risk of bias in all studies was low (Table II, Supplementary Tables IV and V, online only).

## Discussion

This systematic review synthesizes the current evidence with regard to presentation, management, and complications observed in this rare but clinically important injury.

### Clinical presentation and mechanism of injury

We emphasize the need for a high index of suspicion for the accurate and prompt diagnosis of associated vascular injury in patients presenting with proximal humerus fractures. This is especially significant given the time-critical nature, with joint orthopedic, plastic, and vascular surgery guidelines advocating management within 4 hours.^9^ Furthermore, increased risk of limb loss is associated with delays, leading to prolonged ischemic time.^10,56^ More than half of proximal humerus fractures associated with vascular injury occurred after a fall from a standing height, therefore highlighting the importance of considering such an injury even in the absence of a high-energy mechanism. Although the importance of the management of major trauma is reflected in guidelines for the management of vascular trauma and fracture-related arterial injury,^9,57^ it is also vital to emphasize the incidence of these significant injuries in low-energy trauma. This is particularly meaningful in the context of proximal humerus fractures, with increasing incidence of fragility fractures of the humerus due to an aging population.^58^

A cool, pale limb was present in the majority of cases, with 13% also recorded as having an increase in capillary refill time. Interestingly, 13% of all cases did not have any of these clinical features, suggesting that reliance on these clinical features alone may miss an associated vascular injury in more than 1 in 10 cases. In addition, concurrent neurological injury was present in the majority of cases, suggesting that we should not be falsely reassured by a normal capillary refill time when these other features, particularly neurological injury, are present. This is implied in the current BOAST guidelines, which advises “associated nerve injury should be presumed until disproven.”^9^ Our findings, however, emphasize the importance of this also as an indicator of associated vascular injury and should act as a further prompt to consider the presence of vascular injury. Indeed, concomitant brachial plexus injury has been identified as a risk factor for associated axillary artery injury in one retrospective study.^7^ Similarly, an absent or altered pulse was not always present in individuals with an associated vascular injury, and therefore a normal pulse does not necessarily exclude this.

### Imaging

In this review, preoperative imaging to delineate vascular injury was variable with mixed imaging modalities used. Several cases proceeded to surgical management without prior vascular imaging, of which several subsequently required intraoperative imaging. In the current joint guidelines, CT angiography is the only imaging modality discussed and is advised to be undertaken immediately after a CT trauma scanogram.^9^ This, however, also implies the assumption of major trauma as the mechanism of injury. Our review emphasizes the consideration of appropriate imaging in lower mechanism injuries. In the context of general peripheral vascular trauma, CT angiography is reported to be the mainstay of imaging used for the diagnosis of vascular injury.^57^

### Management

Vascular injuries associated with proximal humerus fractures were predominantly arterial in nature and most commonly treated with interposition graft, with preference for venous over prosthetic, followed by primary repair. This is in keeping with current guidelines, which state that these are preferable to the use of bypass grafts.^9^ Vein grafts were traditionally thought to have higher patency and reduced infection rates;^59^ however, more recent literature on the use of interposition grafts in the context of trauma suggests that there may be no difference, albeit these data are retrospective in nature.^60^ Of note, the vein interposition graft required conversion to prosthetic in some cases in this review, and the possible need for conversion should be considered. The use of shunts, however, was only reported in a single case. This is surprising given that most cases underwent fracture fixation followed by vascular repair, and guidelines recommend the use of arterial shunts when rapid definitive restoration of flow cannot be achieved in order to reduce ischemic time and subsequent limb loss.^9,56^ However, similar findings are reported in another study assessing the use of temporary shunts in civilian trauma.^61^ Nevertheless, it is key to highlight the availability and importance of the use of arterial shunt during fracture fixation to restore flow before definitive repair.

Proximal humerus fractures are common injuries associated with increasing age and frailty,^58^ as well as in high-energy trauma in younger patients. Male sex, open fracture, concomitant brachial plexus injury, and atherosclerosis have been identified as risk factors for arterial injury in proximal humerus fractures,^7^ the presence of which is an indication for the surgical management of these otherwise commonly conservatively-managed fractures. The heterogeneity seen in the surgical management of proximal humerus fractures reflects that of patient comorbidities, functional status, and observed fracture pattern. In this review, associated vascular injury occurred in a variety of fracture patterns, most commonly in two-part fractures, and was managed most commonly with open reduction internal fixation. The literature suggests that decision-making between open reduction internal fixation and arthroplasty, either hemiarthroplasty or reverse shoulder arthroplasty, should be largely dictated by fracture patterns, degree of displacement, and patient factors, with older and highly comorbid patients more frequently undergoing hemiarthroplasty.^62,63^

### Strengths and limitations

The evidence regarding lower limb arterial injuries associated with fracture has been summarized to date and resulted in clinical guidance available to aid decision-making.^56^ Upper limb arterial injuries, however, are known to differ from lower limb injuries in terms of management and complications.^59^ Despite this, upper limb fractures with associated vascular injuries are less established in the literature, particularly in predominantly low-energy injuries, such as proximal humerus fractures, compared with high-energy major trauma. Our review attempts to address and discuss this in the context of the current multispecialty guidelines. The overall aim is to aid clinicians across specialties involved in the surgical management of these injuries in the decision-making process, as well as those assessing these injuries at initial presentation. This systematic review, conducted according to PRISMA guidelines,^13^ presents the current evidence that was found to have low risk of bias. We do acknowledge, however, several potential limitations within this. For example, the included studies are case reports and series, representing a lower level of evidence than other retrospective or prospective study designs. As such, despite the low risk of bias suggesting that these studies were well conducted, the evidence collated from this must be interpreted accordingly, and caution used when extrapolating. Therefore, although we describe the reported complications, no consecutive observational studies were identified to be able to truly comment on rates of outcomes and complications with confidence. Rates of amputation, however, have previously been reported to be lower in upper limb compared with lower limb arterial injuries, in keeping with the low rate we observed.^59^ Furthermore, although reduced ischemic time has been shown to be vital in terms of limb salvage within lower limb arterial injuries,^56^ this was not sufficiently reported to comment on in this study. For this reason, this review intends to describe and evaluate reported assessment and management, discussed alongside current guidelines, to facilitate early identification and aid surgical decision-making in these injuries.

## Conclusions

Vascular injuries are seen in a wide range of proximal humerus fracture patterns, but most commonly in displaced two-part proximal humerus fractures. Vascular injury should be particularly considered in the presence of nerve injury with vascular repair predominantly performed by interposition graft or primary repair and fracture management most commonly by open reduction internal fixation. The use of arterial shunts may be vital to restore flow while fracture fixation occurs before definitive vascular repair. Proximal humerus fractures with associated vascular injury occur most commonly in the older adults after low-energy trauma such as a fall from a standing height. A high index of suspicion is needed as not all injuries present with classical ischemic symptoms and these injuries carry a significant burden of morbidity.

## Supplementary Material

Supplementary

## Figures and Tables

**Fig:**
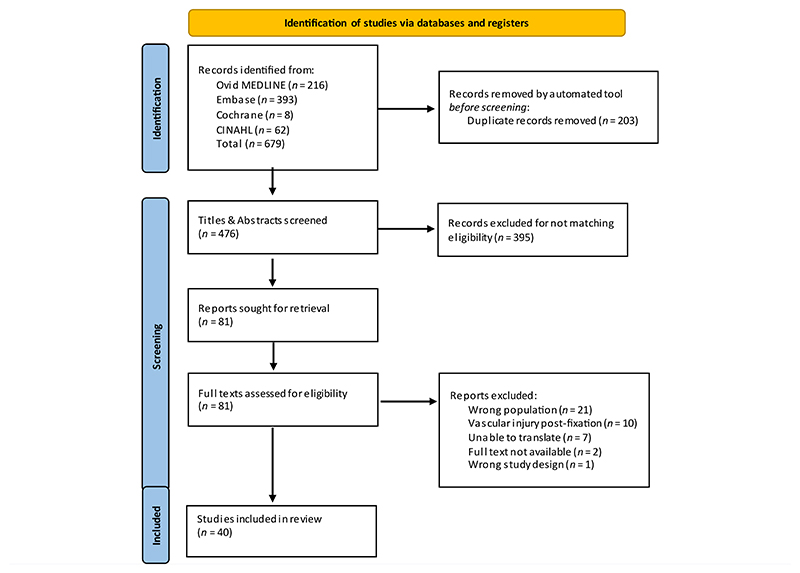
The Preferred Reporting Items for Systematic Reviews and Meta-analyses (PRISMA)^13^ flow diagram for studies reporting the outcome of proximal humerus fractures and associated vascular injury.

**Table I:** Clinical presentation of individuals included per study on proximal humerus fractures associated with vascular injury

Study details			Patient demographics				Vascular presentation						Orthopedic presentation	
First author	Year		Age	Sex	Injury		Cool	Pale	Increased CRT	Pulse	Doppler		Neurology affected	Open fracture
Bucci^18^	2017		85	F	Fall—standing height		N	N	N	Normal	NA		Motor	N
Cawich^19^	2015		68	F	High-energy blunt trauma—fall down stairs		Y	Y	–	Reduce	Absent		–	N
Cotman^20^	2017		79	M	Fall—standing height		–	Y	–	Absent	Absent		Both	N
Di Giacomo^21^	2021		70	F	Fall—standing height		N	N	N	Absent	Absent		NA	N
Githens^22^	2018		57	M	High-energy blunt		Y	–	–	Absent	–		Both	N
Goyal^23^	2014		59	F	Blunt trauma		–	–	Present	Absent	Monophasic		–	N
Hayes^24^	1983		42	F	Fall—standing height		Y	–	–	Absent	Absent		Sensory	N
Hegde^25^	2021		48	M	High-energy blunt trauma		–	–	–	Normal	Present		Both	N
Hofman^26^	2011		92	F	Fall—standing height		Y	Y	Y	Reduce	Monophasic		–	N
			48	M	Fall—standing height		–	–	–	Absent	Absent		Sensory	N
Irimia^27^	2019		58	M	High-energy blunt trauma		Y	Y	Y	Absent	Absent		Both	N
Isawa^28^	2015		82	M	Fall—standing height		Y	Y	–	Absent	–		Sensory	N
Kanda^29^	2020		66	F	Fall—standing height		–	–	–	Reduce	–		Motor	N
Karita^30^	2018		88	F	Fall—standing height		–	Y	–	Normal	–		Motor	N
Kese^31^	2011		51	M	Fall—standing height		–	–	–	Absent	Absent		Both	N
Kurnaz^32^	2018		80	F	Atraumatic		–	–	–	Absent	–		–	N
Lam^33^	2005		85	M	High-energy blunt		Y	Y	Y	Absent	–		Sensory	N
Laverick^34^	1990		65	F	Fall—standing height		Y	Y	–	Absent	–		–	N
Lim^35^	1987		91	F	Fall—standing height		N	–	–	Absent	–		–	N
			79	M	Fall—standing height		Y	–	–	Reduce	–		–	N
			77	M	High-energy blunt trauma—fall downstairs		Y	Y	–	Reduce	40 mm Hg (brachial = 150 mm Hg)		–	N
Lin^36^	2007		29	M	High-energy blunt trauma—fall from 30 ft		Y	Y	N	Absent	–		Both	N
Manak^37^	1996		77	M	Fall—standing height		Y	–	–	Absent	–		–	N
McLaughlin^38^	1998		77	F	Fall—standing height		–	N	N	Absent	–		–	N
Modi^39^	2008		41	F	Fall—standing height		Y	N	N	Absent	Monophasic		Sensory	N
Mouzopoulos^40^	2008		78	F	Fall—standing height		Y	Y	–	Absent	–		Motor	N
Mouzopoulos^41^	2008		45	M	Fall—standing height		N	N	N	Absent	–		–	N
			65	F	High-energy blunt trauma—fall downstairs		Y	Y	–	Absent	–		Motor	N
Naouli^42^	2016		24	M	High-energy blunt trauma		Y	Y	–	Absent	–		–	N
Palanisamy^43^	2017		20	M	High-energy blunt trauma		–	–	–	Reduce	–		Sensory	N
Paley^44^	1986		60	M	High-energy blunt trauma		Y	–	–	–	–		–	N
Palm^45^	2013		38	W	High-energy blunt trauma		N	N	N	Absent	Monophasic		Both	N
Peters^46^	2017		56	M	High-energy blunt trauma		Y	–	–	Absent	–		Sensory	N
			96	F	Fall—Standing height		–	–	–	Absent	–		–	Y
			81	M	High-energy blunt trauma		–	–	–	Normal	–		Sensory	N
			85	F	Fall—standing height		–	–	–	Reduce	–		–	N
			94	F	High-energy blunt trauma		–	–	–	Reduce	–		–	N
Puri^47^	1985		77	W	Fall—standing height		Y	Y	–	Absent	–		Both	N
Razaeian^48^	2018		76	M	Fall—standing height		–	Y	Y	Absent	–		–	N
Seagger^49^	2009		88	F	Fall—standing height		N	–	N	Absent	Absent		Motor	N
Smyth^50^	1969		86	F	Fall—standing height		Y	Y	–	Absent	–		–	N
Stromqvist^51^	1987		79	M	Fall—standing height		–	–	–	Normal	–		–	N
			60	F	High-energy blunt trauma		–	–	–	Absent	–		Both	N
			54	M	High-energy blunt trauma		–	–	–	Normal	–		–	N
Sukeik^52^	2009		74	F	Fall—standing height		Y	Y	Y	Absent	Absent		–	N
Theodorides^16^	1976		30	–	High-energy blunt trauma		–	–	–	Absent	–		–	N
			68	–	Fall—standing height		Y	Y	–	Absent	–		–	N
Thorsness^53^	2014		60	F	Fall—standing height		Y	–	–	Absent	–		Both	N
Yagubyan^17^	2004		68	–	Fall—standing height		Y	–	–	Absent	Absent		Both	N
			69	–	High-energy blunt trauma		Y	–	–	Absent	Present		Motor	N
			78	F	Fall—standing height		Y	Y	Y	Absent	Monophasic		Both	N
Zhang^54^	2012		22	M	Fall—standing height		Y	Y	Y	Absent	–		Both	N
			45	M	High-energy blunt trauma		Y	–	–	–	–		Sensory	N
			47	M	High-energy blunt trauma		N	–	–	Reduce	–		–	N
Zuckerman^55^	1983		64	M	Fall—standing height		Y	Y	–	Absent	Absent		Motor	N

*CRT*, Capillary refill time; *F*, female; *M*, male; *N*, no; *NA*, not applicable; *Y*, yes.

**Table II:** Management of individuals included per study on proximal humerus fractures associated with vascular injury

Study details					Injury			Management			Quality
First author	Year	Country	Design		Vascular injury	Fracture pattern		Fracture	Vascular		JBI score
Bucci^18^	2017	Spain	Case report		Arterial	Nondislocated, displaced Neer 2		ORIF	Interposition vein graft → prosthetic bypass		10/10
Cawich^20^	2015	Trinidad and Tobago	Case report		Arterial	Nondislocated surgical neck of humerus		K-wire	Interposition vein graft → interposition prosthetic graft		9/10
Cotman^20^	2017	United States	Case report		Arterial	Dislocated, Neer 2		ORIF	Vein bypass		9/10
Di Giacomo^21^	2021	Italy	Case report		Arterial	Dislocated, displaced, Neer 4		Reverse shoulder arthroplasty	Vein bypass		10/10
Githens^22^	2018	United States	Case report		Arterial and venous	Dislocated, displaced, Neer 4		ORIF → shoulder hemiarthroplasty	Primary repair (+thrombectomy)		9/10
Goyal^23^	2014	India	Case report		Arterial	–		ORIF	Interposition vein graft		9/10
Hayes^24^	1983	United States	Case report		Arterial	Nondislocated, minimally displaced		K-wire	Primary repair		8/10
Hegde^25^	2021	India	Case report		Arterial	Nondislocated, displaced Neer 4		ORIF	Primary repair (+thrombectomy)		9/10
Hofman^26^	2011	Germany	Case series		Arterial	Dislocated, displaced type II-A3		ORIF	Endovascular (+thrombectomy)		6/9
					Arterial	Dislocated, displaced type II-B3		Hemiarthroplasty	Endovascular stent		–
Irimia^27^	2019	Spain	Case report		Arterial	Dislocated, displaced Neer 4		ORIF	Interposition vein graft (+thrombectomy)		9/10
Isawa^28^	2015	Japan	Case report		Arterial	Displaced Neer 3		ORIF	Endovascular stent		10/10
Kanda^29^	2020	Japan	Case report		Arterial	Nondislocated, displaced Neer 4		Shoulder arthroplasty	Endovascular stent		10/10
Karita^30^	2018	Japan	Case report		Arterial	Nondislocated, displaced Neer 4		Removal of humerus head	Prosthetic bypass		9/10
Kese^31^	2011	Turkey	Case report		Arterial	Nondislocated, displaced, surgical neck of humerus		K-wire	Ligation		9/10
Kurnaz^32^	2018	Turkey	Case report		Arterial	Dislocated, displaced Neer 4		Resection arthroplasty	Interposition prosthetic graft		8/10
Lam^33^	2005	United Kingdom	Case report		Arterial	Nondislocated, displaced Neer 2		ORIF	Conservative		8/10
Laverick^34^	1990	United Kingdom	Case report		Arterial	Subluxed, displaced surgical neck of humerus		Hemiarthroplasty	Interposition prosthetic graft		9/10
Lim^35^	1987	United States	Case series		Arterial	Nondislocated, displaced surgical neck of humerus		Neer proximal humerus replacement	Primary repair		6/9
					Arterial	Nondislocated, displaced surgical neck of humerus		ORIF	Interposition vein graft		–
					Arterial	Nondislocated, displaced surgical neck of humerus		Rods	Primary repair		–
Lin^36^	2007	China	Case report		Arterial	Dislocated greater tuberosity		Reduction	Conservative		10/10
Manak^37^	1996	Czech Republic	Case report		Arterial	Nondislocated, displaced Neer 3		Conservative	Interposition prosthetic graft		10/10
McLaughlin^38^	1998	United States	Case report		Arterial	Dislocated, displaced Neer 3		Humerus head prosthesis	Primary repair (+thrombectomy)		9/10
Modi^39^	2008	United Kingdom	Case series		Arterial	Dislocated, displaced Neer 3		K-wire	Patch repair		6/9
Mouzopoulos^40^	2008	Greece	Case report		Arterial	–		Reduction and bone sutures	Interposition vein graft		10/10
Mouzopoulos^41^	2008	Greece	Case series		Arterial	Displaced		K-wire	Ligation		6/9
					Arterial	Nondislocated, displaced Neer 2		Stabilized—bone sutures	Interposition vein graft		–
Naouli^42^	2016	Morocco	Case report		Arterial	Nondislocated, displaced surgical neck of humerus		–	Interposition vein graft		9/10
Palanisamy^43^	2017	India	Case report		Arterial	Nondislocated, displaced Neer 4		K-wires → ORIF	Conservative		8/10
Paley^44^	1986	Canada	Case report		Arterial	Dislocated, displaced greater tuberosity		Reduction	Conservative		8/10
Palm^45^	2013	United States	Case report		Arterial	Nondislocated, minimally displaced comminuted head of humerus and surgical neck		ORIF	Conservative		9/10
Peters^46^	2017	United States	Case series		Arterial	Nondislocated, displaced Neer 3		Open reduction	Interposition prosthetic graft		6/9
					Arterial	Dislocated, displaced Neer 3		Hemiarthroplasty	Interposition prosthetic graft (+shunt)		–
					Arterial	Dislocated, displaced greater tuberosity		Hemiarthroplasty	Conservative		–
					Arterial	Dislocated, displaced surgical neck of humerus		Hemiarthroplasty	Endovascular stent		–
					Arterial	Dislocated, displaced Neer 3		ORIF	Interposition prosthetic graft		–
Puri^47^	1985	United Kingdom	Case report		Arterial	Nondislocated, displaced surgical neck of humerus		ORIF	Primary repair		10/10
Razaeian^48^	2018	Germany	Case report		Arterial	Nondislocated, displaced Neer 4		Head replacement	Interposition vein graft (+thrombectomy)		9/10
Seagger^49^	2009	United Kingdom	Case report		Arterial	Nondislocated, displaced Neer 2		ORIF	Vein patch (+thrombectomy)		9/10
Smyth^50^	1969	United Kingdom	Case report		Arterial	Nondislocated, displaced Neer 3		Not reported	Primary repair (+clot aspiration)		9/10
Stromqvist^51^	1987	Sweden	Case series		Arterial	Displaced		Rush pins	Primary repair		6/9
					Arterial	Nondislocated, displaced humerus head split and neck		ORIF	Primary repair		–
					Arterial	Nondislocated, displaced Neer 3		ORIF	Patch repair		–
Sukeik^52^	2009	United Kingdom	Case report		Arterial	Nondislocated, minimally displaced surgical neck of humerus		Conservative	Conservative		10/10
Theodorides^16^	1976	Nether-lands	Case series		Arterial	Nondislocated, displaced surgical neck of humerus		Nail	Interposition vein graft		6/9
					Arterial	Nondislocated, displaced, surgical neck of humerus		Conservative	Interposition vein graft		–
Thorsness^53^	2014	United States	Case series		Venous	Dislocated, Neer 3		ORIF	Conservative		7/9
Yagubyan^17^	2004	United States	Case series		Arterial and venous	Displaced head of humerus		ORIF	Primary repair		7/9
					Arterial	Dislocated, displaced head of humerus and surgical neck of humerus		Hemiarthroplasty	Interposition vein graft		
					Arterial	Nondislocated, displaced surgical neck of humerus		K-wire	Interposition vein graft		–
Zhang^54^	2012	China	Case series		Arterial	Nondislocated, displaced surgical neck of humerus		ORIF	Interposition vein graft		6/9
					Arterial	Nondislocated, displaced surgical neck of humerus		ORIF	Conservative		–
					Arterial	Nondislocated, displaced surgical neck of humerus		Reduced and fixed	Conservative		–
Zuckerman^55^	1983	United States	Case series		Arterial	Displaced Neer 2		Rods	Thrombectomy and interposition vein graft		6/9

*JBI*, Joanna Brigg’s Institute; *ORIF*, open reduction internal fixation.

**Table III:** Postoperative outcome of individuals included per study on proximal humerus fractures associated with vascular injury

Study details			Clinical					Imaging	
First author	Year		Pulse	Sensation	Motor	ROM		Vascular	Fracture
Bucci^18^	2017		Present	–	Postganglionic brachial plexopathy	–		N	N
Cawich^19^	2015		Present	–	–	Full		N	N
Cotman^20^	2017		–	Reduced: ulnar border	–	No motion elbow/wrist/ hand		N	X ray
Di Giacomo^21^	2021		Present	–	–	Quick Dash score 22; ROM 110° active elevation and 100° active abduction		–	X ray
Githens^22^	2018		Present	–	Complete brachial plexopathy	–		–	–
Goyal^23^	2014		Present	–	–	–		N	N
Hayes^24^	1983		Present	Normal	Normal	Normal		N	N
Hegde^25^	2021		–	Normal	Normal	Good		N	N
Hofman^26^	2011		–	–	Deficit hand muscles—improved	Intact		N	–
Irimia^27^	2019		–	–	–	50° flexion, 40° abduction		Doppler	X ray
Isawa^28^	2015		–	–	–	–		N	N
Kanda^29^	2020		Present	Normal	Normal	Normal		N	N
Karita^30^	2018		Present	–	Arm paralysis	–		N	N
Kese^31^	2011		–	–	Radial nerve dysfunction –normal after 3 months	Normal		N	N
Kurnaz^32^	2018		Present	Normal	Normal	Normal		N	N
Lam^33^	2005		–	–	–	–		–	
Laverick^34^	1990			Normal	Normal	Normal		DSA	N
Lim^35^	1987		Present	–	–	–		N	N
			Present	–	–	Functional		N	X ray
			–	–	–	Flexion 60°, abduction 60°, external rotation 20°		N	N
Lin^36^	2007			Numb: ulnar 2 fingers	4/5 deltoid power, minimal intrinsic muscle weakness hand	Active abduction 110° with 120° of forward elevation		N	X ray
Manak^37^	1996		–	–	–	Active flexion shoulder 70°, extension 40°, abduction 40°		Doppler	N
McLaughlin^38^	1998		Present	–	Neer shoulder score at 1 year = 83	–		N	N
Modi^39^	2008		–	–	–	–		N	Y
Mouzopoulos^40^	2008		–	–	–	–		N	
Mouzopoulos^41^	2008		–	–	–	Functional		Doppler	N
			–	–	–	Functional		Doppler	Y
Naouli^42^	2016		Present	Normal	Normal	Normal		N	N
Palanisamy^43^	2017		–	–	–	Normal		N	X ray
Paley^44^	1986		–	–	Deltoid 4/5	Normal		N	–
Palm^45^	2013		–	Reduced: lateral and medial forearm	–	Normal		N	N
Peters^46^	2017		–	–	Improved	Improved		–	–
			Present	Normal	Normal	Passive flexion 120°, active flexion and abduction 30°		–	Y
			–	–	–	Forward flexion 85°, external flexion 15°, and internal rotation to L5		–	–
			Present	–	–	Abduction 45°		–	X ray
Puri^47^	1985		Present	–	–	–		–	–
Razaeian^48^	2018		Present	–	Plexopathy	Shoulder function 10° and Constant Shoulder Score 17/100		N	X ray
Seagger^49^	2009		–	–	4/5 elbow flexion, extension 2/5	–		N	X ray
Smyth^50^	1969		–	–	–	–		N	–
Stromqvist^51^	1987		Present	–	Palsy of radial branch	–		–	–
			–	–	50% reduction grip strength	Reduction in arm mobility		N	–
Sukeik^52^	2009		Present	–	–	Good		–	–
Theodorides^16^	1976		–	–	–	–		DSA	–
			–	–	–	Restricted abduction		DSA	–
Thorsness^53^	2014		–	–	Complex brachial plexopathy	–		Doppler	N
Yagubyan^17^	2004		Present	Regained C5/6	–	–		–	–
			Present	–	Functional use for light grasping	–		–	–
			Present	–	Weakness wrist and finger extension, and handgrip	–		DSA	–
Zhang^54^	2012		–	Y	Weakness of wrist and finger extension	–		CTA	–
			–	Numb: 2 ulnar fingers	–	–		–	–
Zuckerman^55^	1983		Present	–	Posterior cord lesion	–		Doppler	X ray

*CTA*, Computed tomography angiogram; *DSA*, digital subtraction angiography; *N*, no; *ROM*, range of motion; *Y*, yes.

## References

[1] Pencle FJ, Gossman WG (2022). Proximal humerus fracture.

[2] Court-Brown CM, Caesar B (2006). Epidemiology of adult fractures: a review. Injury.

[3] Relvas Silva M, Linhares D, Leite MJ (2022). Proximal humerus fractures: epidemiology and trends in surgical management of hospital-admitted patients in Portugal. JSES Int.

[4] Handoll HHG, Elliott J, Thillemann TM, Aluko P, Brorson S (2022). Interventions for treating proximal humeral fractures in adults. Cochrane Database Syst Rev.

[5] Rangan A, Handoll H, Brealey S (2015). Surgical vs nonsurgical treatment of adults with displaced fractures of the proximal humerus: the PROFHER randomized clinical trial. JAMA.

[6] Jawa A, Burnikel D (2016). Treatment of proximal humeral fractures: a critical analysis review. JBJS Rev.

[7] Menendez ME, Ring D, Heng M (2015). Proximal humerus fracture with injury to the axillary artery: a population-based study. Injury.

[8] Bravman JT, Ipaktchi K, Biffl WL, Stahel PF (2008). Vascular injuries after minor blunt upper extremity trauma: pitfalls in the recognition and diagnosis of potential “near miss” injuries. Scand J Trauma Resusc Emerg Med.

[9] British Orthopaedic Association Trauma Committee (2021). British orthopaedic assication standard for trauma (BOAST): Diagnosis & management of arterial injuries associated with extremity fractures and dislocations. Injury.

[10] Alarhayem AQ, Cohn SM, Cantu-Nunez O, Eastridge BJ, Rasmussen TE (2019). Impact of time to repair on outcomes in patients with lower extremity arterial injuries. J Vasc Surg.

[11] Neer CS (2002). Four-segment classification of proximal humeral fractures: purpose and reliable use. J shoulder Elb Surg.

[12] Marongiu G, Leinardi L, Congia S, Frigau L, Mola F, Capone A (2020). Reliability and reproducibility of the new AO/OTA 2018 classification system for proximal humeral fractures: a comparison of three different classification systems. J Orthop Traumatol.

[13] Page MJ, McKenzie JE, Bossuyt PM (2021). The PRISMA 2020 statement: an updated guideline for reporting systematic reviews. BMJ.

[14] Institute JB (2017). Checklist for Case Series Critical Appraisal.

[15] McHugh ML (2012). Interrater reliability: the kappa statistic. Biochem Medica.

[16] Theodorides T, de Keizer C (1976). Injuries of the axillary artery caused by fractures of the neck of the humerus. Injury.

[17] Yagubyan M, Panneton JM (2004). Axillary artery injury from humeral neck fracture: a rare but disabling traumatic event. Vasc Endovascular Surg.

[18] Bucci G, Lucar-Lopez G, Sanchez-Gonzalez J, Malagelada F, Palencia Lopez G, Guevara-Noriega KA (2017). Axillary artery injury and brachial plexus palsy as a complication of proximal humerus fractures. J Orthop.

[19] Cawich SO, Harnarayan P, Budhooram S, Naraynsingh V (2014). Axillary artery injury accompanying humeral neck fracture. Int J Angiol.

[20] Cotman SJ, Trinh TQ, Vincent S, Backes JR (2017). Proximal humerus fracture-dislocation with laceration of the axillary artery: a case report. Iowa Orthop J.

[21] Di Giacomo LM, Marzano F, Zaganelli A (2021). Two stage treatment of a proximal humeral fracture-dislocation with vascular injury: case report of a multidisciplinary approach. Trauma case reports.

[22] Githens M (2018). Life-threatening hemorrhage from an unrecognized axillary vein injury during treatment of a proximal humeral fracture-dislocation with a known axillary artery injury: a case report. JBJS case Connect.

[23] Goyal VD, Sharma V, Kalia S, Sehgal M (2014). Axillary artery injury caused by fracture of humerus neck and its repair using basilic vein graft. Case Rep Surg.

[24] Hayes JM, Van Winkle GN (1983). Axillary artery injury with minimally displaced fracture of the neck of the humerus. J Trauma.

[25] Hegde N, Kundangar RS, Nishanth A, Bhat SN (2021). Disappearing pulse: proximal humerus fracture with acute thrombosis of axillary artery. BMJ Case Rep.

[26] Hofman M, Grommes J, Krombach GA, Schmidt-Rohlfing B (2011). Vascular injury accompanying displaced proximal humeral fractures: two cases and a review of the literature. Emerg Med Int.

[27] Irimia JC, Garrido DG, Bisaccia M, Rollo G, Meccariello L, Tome-Bermejo F (2019). Comminuted bilateral simultaneous fracture and luxation of the proximal humerus with an injury to the right axillary artery. J Orthop case reports.

[28] Isawa T, Suzuki K, Abe H (2015). Upper limb salvage with endovascular treatment of acute axillary artery occlusion secondary to proximal humeral fracture. Vasc Dis Manag.

[29] Kanda D, Imagama I, Imoto Y, Ohishi M (2021). Bidirectional endovascular treatment for axillary artery injury secondary to proximal humerus fracture: a case report. Eur Hear journal Case reports.

[30] Karita Y, Kimura Y, Sasaki S, Nitobe T, Tsuda E, Ishibashi Y (2018). Axillary artery and brachial plexus injury secondary to proximal humeral fractures: a report of 2 cases. Int J Surg Case Rep.

[31] Kese S, Ege A, Turhan E, Songür Bayar A, Akça MK (2011). Axillary artery entrapment following proximal humeral fracture: a case report and review of literature. Eur J Orthop Surg Traumatol.

[32] Kurnaz R, Ikizler M, Ozbayburtlu M, Gunes T (2018). A limb-threatening long arterial dissection caused by humerus neck fracture: a case report. Malaysian Orthop J.

[33] Lam F, Kurta I, Hussain S (2005). Axillary artery injury following a closed fracture of the proximal humerus. J Diagnostic Radiogr Imaging.

[34] Laverick MD, D’Sa AA, Kirk SJ, Mollan RA (1990). Management of blunt injuries of the axillary artery and the neck of the humerus: case report. J Trauma.

[35] Lim EV, Day LJ (1987). Thrombosis of the axillary artery complicating proximal humeral fractures. A report of three cases. J Bone Joint Surg Am.

[36] Lin C-Y, Chen S-J, Yu C-T, Chang I-L (2007). Simultaneous bilateral anterior fracture dislocation of the shoulder with neurovascular injury: report of a case. Int Surg.

[37] Manak P, Klein J (1996). Axillary artery injury in closed fracture of the humeral neck. Acta Univ Palacki Olomuc Fac Med.

[38] McLaughlin JA, Light R, Lustrin I (1998). Axillary artery injury as a complication of proximal humerus fractures. J shoulder Elb Surg.

[39] Modi CS, Nnene CO, Godsiff SP, Esler CNA (2008). Axillary artery injury secondary to displaced proximal humeral fractures: a report of two cases. J Orthop Surg.

[40] Mouzopoulos G, Lasanianos N, Mouzopoulos D, Batanis G, Tzurbakis M, Georgilas I (2008). Acute renal failure due to rhabdomyolysis after proximal humerus fracture associated with axillary artery rupture. Int Urol Nephrol.

[41] Mouzopoulos G, Lassanianos N, Mouzopoulos D, Tzurbakis M, Georgilas I (2008). Axillary artery injury associated with proximal humerus fractures. Vasa.

[42] Naouli H, Benfor B, Jiber H, Bouarhroum A (2016). Axillary artery injury from a closed humeral neck fracture: a case report. J Mal Vasc.

[43] Palanisamy JV, Vaithilingam A, Das S, Trikha V (2017). Proximal humerus fracture dislocation leading to axillary artery injury in an young adult: case report of an unusual presentation. J Clin Orthop trauma.

[44] Paley D, Love TR, Malcolm BW (1986). Bilateral anterior fracture dislocation of the shoulder. With brachial plexus and axillary artery injury. Orthop Rev.

[45] Palm DS, Parikh PP, Schoonover B, Lebamoff D, McCarthy MC (2013). Axillary arterial entrapment and brachial plexus injury due to proximal humeral fracture. Inj Extra.

[46] Peters RM, Menendez ME, Mellema JJ, Ring D, Smith RM (2017). Axillary artery injury associated with proximal humerus fracture: a report of 6 cases. Arch bone Jt Surg.

[47] Puri R, Clark J, Corkery PH (1985). Axillary artery damage following a closed fracture of the neck of the humerus—a case report. Injury.

[48] Razaeian S, Rustum S, Sonnow L, Meller R, Krettek C, Hawi N (2018). Axillary artery dissection and secondary thrombosis after closed proximal humerus fracture-a rare interdisciplinary challenge. Eur J Mol Clin Med.

[49] Seagger RM, Kitson J (2009). A rare combination of an axillary artery and brachial plexus injury due to a proximal humeral fracture. Int J Shoulder Surg.

[50] Smyth EH (1969). Major arterial injury in closed fracture of the neck of the humerus. Report of a case. J Bone Joint Surg Br.

[51] Stromqvist B, Lidgren L, Norgren L, Odenbring S (1987). Neurovascular injury complicating displaced proximal fractures of the humerus. Injury.

[52] Sukeik M, Vashista G, Shaath N (2009). Axillary artery compromise in a minimally displaced proximal humerus fracture: a case report. Cases J.

[53] Thorsness R, English C, Gross J, Tyler W, Voloshin I, Gorczyca J (2014). Proximal humerus fractures with associated axillary artery injury. J Orthop Trauma.

[54] Zhang Q, Wang S, Tang C, Chen W, Zhang Y, Chen L (2013). Axillary artery lesions from humeral neck fracture: a study in relation to repair. Exp Ther Med.

[55] Zuckerman JD, Flugstad DL, Teitz CC, King HA (1984). Axillary artery injury as a complication of proximal humeral fractures. Two case reports and a review of the literature. Clin Orthop Relat Res.

[56] Glass GE, Pearse MF, Nanchahal J (2009). Improving lower limb salvage following fractures with vascular injury: a systematic review and new management algorithm. J Plast Reconstr Aesthet Surg.

[57] Kobayashi L, Coimbra R, Goes AMOJ (2020). American Association for the Surgery of Trauma-World Society of Emergency Surgery guidelines on diagnosis and management of peripheral vascular injuries. J Trauma Acute Care Surg.

[58] Kumar S, Sonar U, Singh I (2018). Fragility fractures in the upper limb: proximal and distal humerus. Br J Hosp Med.

[59] Klocker J, Bertoldi A, Benda B, Pellegrini L, Gorny O, Fraedrich G (2014). Outcome after interposition of vein grafts for arterial repair of extremity injuries in civilians. J Vasc Surg.

[60] Rehman ZU (2020). Outcomes of popliteal artery injuries repair: autologous vein versus prosthetic interposition grafts. Ann Vasc Surg.

[61] Abou Ali AN, Salem KM, Alarcon LH (2017). Vascular shunts in civilian trauma. Front Surg.

[62] Thorsness R, Iannuzzi J, Noyes K, Kates S, Voloshin I (2014). Open reduction and internal fixation versus hemiarthroplasty in the management of proximal humerus fractures. Geriatr Orthop Surg Rehabil.

[63] Yahuaca BI, Simon P, Christmas KN (2020). Acute surgical management of proximal humerus fractures: ORIF vs. hemiarthroplasty vs. reverse shoulder arthroplasty. J shoulder Elb Surg.

