# Supplementary material for: A systematic review of proximal humerus fractures and associated vascular injuries
Source: JVS Vasc Insights. Author manuscript; Available in PMC 2025 Apr 10. (PMC7617580; doi:10.1016/j.jvsvi.2024.100065)
Supplement: Supplementary [file EMS204036-supplement-Supplementary.docx]

**SUPPLEMENTARY FILES**

**Supplementary Figure 1.** Search Strategy for: Ovid MEDLINE(R) ALL <1946 to January 11, 2023> (Run 1st February 2023)

1 shoulder fractures/

2 humeral fractures/

3 ((humer* or shoulder*) adj10 (fracture* or fixat*)).mp.

4 ((humer* or shoulder*) adj10 (break* or broke* or crack*)).mp.

5 ((humer* or shoulder*) adj10 comminute*).mp.

6 or/2-5

7 (proximal or neck*1 or sub?capital).tw.

8 6 and 7

9 Shoulder/ or Shoulder Joint/

10 Fractures, Bone/

11 9 and 10

12 1 or 8 or 11

13 (vascular* adj4 (injur* or damag*)).tw,kw,kf.

14 ("blood vessel*" adj4 (injur* or damag*)).tw,kw,kf.

15 (arter* adj4 (injur* or damag*)).tw,kw,kf.

16 (limb* adj4 salvag*).tw,kw,kf.

17 amput*9.tw,kw,kf.

18 disarticulat*.tw,kw,kf.

19 re-vascular*9.tw,kw,kf.

20 revascular*9.tw,kw,kf.

21 Vascular System Injuries/

22 Axillary Artery/ab, in, pp, su [Abnormalities, Injuries, Physiopathology, Surgery]

23 Brachial Artery/ab, in, pp, su [Abnormalities, Injuries, Physiopathology, Surgery]

24 Limb Salvage/

25 amputation/

26 disarticulation/

27 or/13-26

28 12 and 27

**Supplementary Table 1:** Study eligibility criteria.

| **Inclusion criteria** | **Exclusion criteria** |
| --- | --- |
| Patients ≥ 18 years | Humerus fractures without vascular injury |
| Proximal humerus fracture with associated vascular injury | Distal/supra-condylar humerus fractures |
|  | Dislocation of humerus with no associated fracture |
|  | Vascular injury due to fracture manipulation/surgical intervention |
|  | Vascular injuries obtained post-fixation |
|  | Vascular injury after > 24 hours |

**Supplementary Table 2.** Joanna Briggs Institute critical appraisal tools checklist for Case Reports.

| **Questions** | **Yes/No/Unclear/Not applicable** |
| --- | --- |
| 1. Were patient’s demographic characteristics clearly described? |  |
| 1. Was the patient’s history clearly described and presented as a timeline? |  |
| 1. Was the current clinical condition of the patient on presentation clearly described? |  |
| 1. Were diagnostic tests or assessment methods and the results clearly described? |  |
| 1. Was the intervention(s) or treatment procedure(s) clearly described? |  |
| 1. Was the post-intervention clinical condition clearly described? |  |
| 1. Were adverse events (harms) or unanticipated events identified and described? |  |
| 1. Does the case report provide takeaway lessons? |  |

**Supplementary Table 3.** Joanna Briggs Institute critical appraisal tools checklist for Case Series.

| **Questions** | **Yes/No/Unclear/Not applicable** |
| --- | --- |
| 1. Were there clear criteria for inclusion in the case series? |  |
| 1. Was the condition measured in a standard, reliable way for all participants included in the case series? |  |
| 1. Were valid methods used for identification of the condition for all participants included in the case series? |  |
| 1. Did the case series have consecutive inclusion of participants? |  |
| 1. Did the case series have complete inclusion of participants? |  |
| 1. Was there clear reporting of the demographics of the participants in the study? |  |
| 1. Was there clear reporting of clinical information of the participants? |  |
| 1. Were the outcomes or follow up results of cases clearly reported? |  |
| 1. Was there clear reporting of the presenting site(s)/clinic(s) demographic information? |  |
| 1. Was statistical analysis appropriate? |  |

**Supplementary Table 4.** Quality assessment of included studies using the Joanna Briggs Institute risk of bias critical appraisal tool for Case Reports.

| **1st Author** | **Demographics** | **History** | **Condition** | **Tests** | **Intervention** | **Post-intervention** | **Adverse events** | **Lessons** | **Overall** |
| --- | --- | --- | --- | --- | --- | --- | --- | --- | --- |
| Bucci^32^ 2017 | ✓ | ✓ | ✓ | ✓ | ✓ | ✓ | ✓ | ✓ | 10/10 |
| Cawich^33^ 2015 | X | ✓ | ✓ | ✓ | ✓ | ✓ | ✓ | ✓ | 9/10 |
| Cotman^26^ 2017 | X | ✓ | ✓ | ✓ | ✓ | ✓ | ✓ | ✓ | 9/10 |
| Giacomo^25^ 2021 | ✓ | ✓ | ✓ | ✓ | ✓ | ✓ | ✓ | ✓ | 10/10 |
| Githens^30^ 2018 | X | ✓ | ✓ | ✓ | ✓ | ✓ | ✓ | ✓ | 9/10 |
| Goyal^22^ 2014 | ✓ | ✓ | ✓ | X | ✓ | ✓ | ✓ | ✓ | 9/10 |
| Hayes^19^ 1983 | X | ✓ | ✓ | ✓ | X | ✓ | ✓ | ✓ | 8/10 |
| Hegde^34^ 2021 | X | ✓ | ✓ | ✓ | ✓ | ✓ | ✓ | ✓ | 9/10 |
| Irimia^36^ 2019 | X | ✓ | ✓ | ✓ | ✓ | ✓ | ✓ | ✓ | 9/10 |
| Isawa^37^ 2015 | ✓ | ✓ | ✓ | ✓ | ✓ | ✓ | ✓ | ✓ | 10/10 |
| Kanda^38^ 2020 | ✓ | ✓ | ✓ | ✓ | ✓ | ✓ | ✓ | ✓ | 10/10 |
| Karita^39^ 2018 | ✓ | ✓ | ✓ | ✓ | X | ✓ | ✓ | ✓ | 9/10 |
| Keser^40^ 2011 | X | ✓ | ✓ | ✓ | ✓ | ✓ | ✓ | ✓ | 9/10 |
| Kurnaz^15^ 2018 | X | ✓ | X | ✓ | ✓ | ✓ | ✓ | ✓ | 8/10 |
| Lam^41^ 2005 | X | ✓ | X | ✓ | ✓ | ✓ | ✓ | ✓ | 8/10 |
| Laverick^21^ 1990 | X | ✓ | ✓ | ✓ | ✓ | ✓ | ✓ | ✓ | 9/10 |
| Lin^29^ 2007 | ✓ | ✓ | ✓ | ✓ | ✓ | ✓ | ✓ | ✓ | 10/10 |
| Manak^27^ 1996 | ✓ | ✓ | ✓ | ✓ | ✓ | ✓ | ✓ | ✓ | 10/10 |
| McLaughlin^43^ 1998 | X | ✓ | ✓ | ✓ | ✓ | ✓ | ✓ | ✓ | 9/10 |
| Mouzopoulos^45^ 2008 | ✓ | ✓ | ✓ | ✓ | ✓ | ✓ | ✓ | ✓ | 10/10 |
| Naouli^23^ 2016 | X | ✓ | ✓ | ✓ | ✓ | ✓ | ✓ | ✓ | 9/10 |
| Palanisamy^47^ 2017 | X | ✓ | ✓ | X | ✓ | ✓ | ✓ | ✓ | 8/10 |
| Paley^48^ 1986 | X | ✓ | ✓ | X | ✓ | ✓ | ✓ | ✓ | 8/10 |
| Palm^17^ 2013 | X | ✓ | ✓ | ✓ | ✓ | ✓ | ✓ | ✓ | 9/10 |
| Puri^49^ 1985 | ✓ | ✓ | ✓ | ✓ | ✓ | ✓ | ✓ | ✓ | 10/10 |
| Razaeian^50^ 2018 | X | ✓ | ✓ | ✓ | ✓ | ✓ | ✓ | ✓ | 9/10 |
| Seagger^31^ 2009 | X | ✓ | ✓ | ✓ | ✓ | ✓ | ✓ | ✓ | 9/10 |
| Smyth^28^ 1969 | X | ✓ | ✓ | ✓ | ✓ | ✓ | ✓ | ✓ | 9/10 |
| Sukeik*.*^20^ 2009 | ✓ | ✓ | ✓ | ✓ | ✓ | ✓ | ✓ | ✓ | 10/10 |

✓*= Yes; X = No; ? = Unsure; NA = Not applicable*

**Supplementary Table 5.** Quality assessment of included studies using the Joanna Briggs Institute risk of bias critical appraisal tool for Case Series.

| **Study** | **Participation Selection** | | | | | | **Results** | | | | **Overall** |
| --- | --- | --- | --- | --- | --- | --- | --- | --- | --- | --- | --- |
|  | **Criteria** | **Condition** | **Clear Identification** | **Consecutive Inclusion** | **Complete Inclusion** | **Demo-graphics** | **Clinical Info** | **Outcome** | **Site** | **A** |  |
| Hofman^35^ 2011 | ✓ | ✓ | ✓ | **?** | **?** | **X** | ✓ | ✓ | ✓ | **NA** | **6/9** |
| Lim^42^ 1987 | ✓ | ✓ | ✓ | **?** | **?** | ✓ | **X** | ✓ | ✓ | **NA** | **6/9** |
| Modi^44^ 2008 | ✓ | ✓ | ✓ | **?** | **?** | **X** | ✓ | ✓ | ✓ | **NA** | **6/9** |
| Mouzopoulos^46^ 2008 | ✓ | ✓ | ✓ | **?** | **?** | **X** | ✓ | ✓ | ✓ | **NA** | **6/9** |
| Peters^16^ 2017 | ✓ | ✓ | ✓ | **?** | **?** | **X** | ✓ | ✓ | ✓ | **NA** | **6/9** |
| Stromqvist^24^ 1987 | ✓ | ✓ | ✓ | **?** | **?** | **X** | ✓ | ✓ | ✓ | **NA** | **6/9** |
| Theodorides^13^ 1976 | ✓ | ✓ | ✓ | **?** | **?** | **X** | ✓ | ✓ | ✓ | **NA** | **6/9** |
| Thorsness^51^ 2014 | ✓ | ✓ | ✓ | **?** | **?** | ✓ | ✓ | ✓ | ✓ | **NA** | **7/9** |
| Yagubyan^14^ 2004 | ✓ | ✓ | ✓ | **?** | **?** | ✓ | ✓ | ✓ | ✓ | **NA** | **7/9** |
| Zhang^18^ 2013 | ✓ | ✓ | ✓ | **?** | **?** | **X** | ✓ | ✓ | ✓ | **NA** | **6/9** |
| Zuckerman^52^ 1983 | ✓ | ✓ | ✓ | **?** | **?** | **X** | ✓ | ✓ | ✓ | **NA** | **6/9** |
